# Beware the glowing fat: pericarditis pitfall

**DOI:** 10.1093/ehjimp/qyae036

**Published:** 2024-05-09

**Authors:** André Vaz

**Affiliations:** Cardiovascular Radiology, InCor, Av. Dr. Enéas Carvalho de Aguiar, 44, Cerqueira César, São Paulo, São Paulo 05403-900, Brazil

Pericardial inflammation in acute and recurrent pericarditis induces increased vascularity, neutrophil infiltration, and fibrin deposition that, in turn, can be detected by magnetic resonance imaging (MRI) using late gadolinium enhancement (LGE) sequences.^1^ A recent study presented by Cremer *et al*. elucidated the role of cardiac MRI in assessing recurrent pericarditis and its potential impact on rilonacept treatment duration.^2^ The authors aptly demonstrated the significance of pericardial LGE in predicting the frequency and timing of pericarditis recurrence, emphasizing the importance of tailored therapeutic strategies based on MRI findings.

Phase-sensitive inversion recovery sequences with an inversion time selected for optimal myocardial nulling were used to detect and grade pericardial LGE in the RHAPSODY trial.^2^ However, I would like to point out that while LGE sequences are sensitive to inflammatory and fibrotic changes, their original design without fat saturation may inadvertently highlight epicardial fat, mimicking enhancement (*Figure 1A*).^3^ Fat usually exhibits a hyperintense signal during the inversion time optimized to null the myocardial signal. LGE sequences with fat saturation are proving particularly useful in this context (*Figure 1B*), enabling more accurate characterization of true pericardial enhancement (*Figure 1C*). In situations lacking fat-saturated LGE sequences, Dixon-type sequences (*Figure 1D*), though not gated, offer an alternative by subtracting fat signal to detect pericardial enhancement. MRI assessment of the pericardium is becoming increasingly common, and a comprehensive understanding of tissue behaviour across different sequences is critical for accurate interpretation of imaging findings.

**Figure 1:**
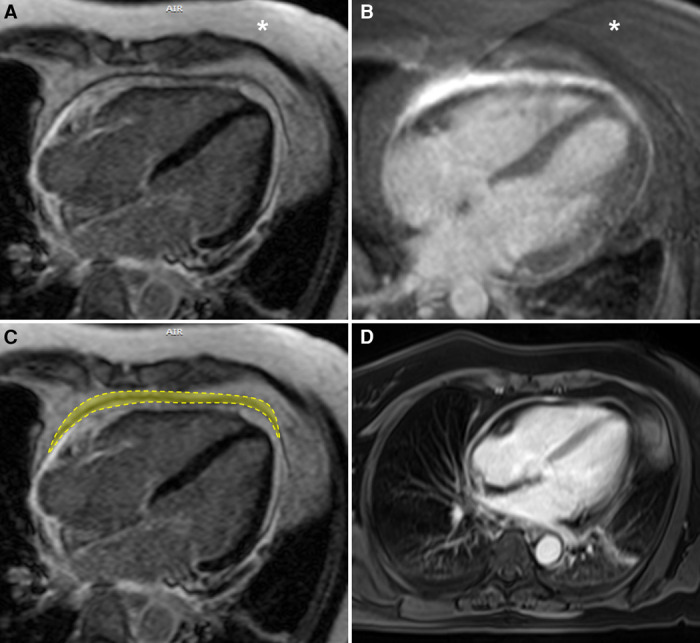
Forty-year-old male presented with acute chest pain. Initial evaluation excluded coronary artery disease. MRI was requested to evaluate for myocarditis and showed pericardial thickening and enhancement. A significant difference can be seen between the areas of hyperintense signal in LGE sequences without and with fat saturation (*A* and *B*, respectively). Note the difference in subcutaneous fat signal intensity (asterisk) in *A* and *B* to differentiate between the non-fat-suppressed and fat-suppressed sequences, respectively. The area of ‘true’ pericardial enhancement (outlined) appears markedly reduced in the sequence without fat saturation (*C*). Pericardial thickening and enhancement can also be characterized in post-contrast non-gated Dixon-type T1-weighted sequence (*D*). Technical parameters: (*A*) non-fat-saturated inversion recovery prepared gradient echo pulse sequence (TR = 881.6 ms, TE = 4.72 ms, FOV = 380, matrix = 232 × 256, flip angle = 25°); (*B*) fat-saturated inversion recovery prepared gradient echo pulse sequence (TR = 630.6 ms, TE = 1.74 ms, FOV = 400, matrix = 232 × 256, flip angle = 20°); and (*D*) dual-echo volumetric interpolated breath-hold examination (VIBE)-Dixon T1-weighted sequence (TR = 6.8 ms, TE1 = 2.39 ms, TE2 = 4.77 ms, FOV = 380, matrix = 256 × 320, flip angle = 8°).

## Lead author biography


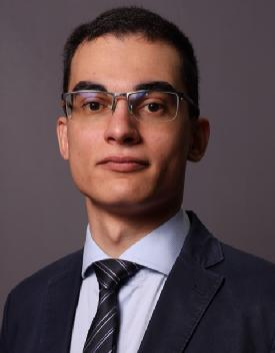


André Vaz is a cardiovascular radiologist based in São Paulo, Brazil. He completed his Fellowship in Cardiovascular CT and MRI at the Instituto do Coração do Hospital de Clínicas da Faculdade de Medicina da Universidade de São Paulo, under the supervision of Carlos Eduardo Rochitte. Prior to this, he earned a Master’s Degree in Internal Medicine from the Universidade Federal do Paraná and completed fellowships in Pediatric Radiology at Hospital Pequeno Príncipe. He obtained his Doctor of Medicine degree from the Universidade Federal de Santa Catarina. He is currently involved in research into congenital heart disease, 3D printing, and genetic cardiomyopathies.
